# Cerebral neurovascular alterations in stable chronic obstructive pulmonary disease: a preliminary fMRI study

**DOI:** 10.7717/peerj.14249

**Published:** 2022-11-14

**Authors:** Zhaohui Peng, Hong Tao Zhang, Gang Wang, Juntao Zhang, Shaowen Qian, Yajun Zhao, Ruijie Zhang, Wei Wang

**Affiliations:** 1 Department of Nuclear Medicine, Central Hospital affiliated to Shandong First Medical University, Jinan, Shandong, China; 2 Department of Medical Imaging, Changzheng Hospital, Shanghai, China; 3 Institute of Ophthalmology, Third Medical Center of PLA General Hospital, Beijing, China; 4 The Second Community Healthcare Service Center of Zhengzhou Road, Luoyang, Henan, China; 5 GE Healthcare, Precision Health Institution, Shanghai, China; 6 Department of Medical Imaging, Jinan Military General Hospital, Jinan, China; 7 Department of Medical Imaging, 71282 Hospital, Baoding, Hebei province, China; 8 Department of Radiology, Qilu Hospital of Shandong University Dezhou Hospital, Dezhou, Shandong Province, China

**Keywords:** Cerebral blood flow, Degree centrality, Functional magnetic resonance, Cognitive impairment, Chronic obstructive pulmonary disease

## Abstract

**Purpose:**

Cognitive impairment (CI) is very common in patients with chronic obstructive pulmonary disease (COPD). Cerebral structural and functional abnormalities have been reported in cognitively impaired patients with COPD, and the neurovascular coupling changes are rarely investigated. To address this issue, arterial spin labeling (ASL) and resting-state blood oxygenation level dependent (BOLD) fMRI techniques were used to determine whether any neurovascular changes in COPD patients.

**Methods:**

Forty-five stable COPD patients and forty gender- and age-matched healthy controls were recruited. Furthermore, resting-state BOLD fMRI and ASL were acquired to calculate degree centrality (DC) and cerebral blood flow (CBF) respectively. The CBF-DC coupling and CBF/DC ratio were compared between the two groups.

**Results:**

COPD patients showed abnormal CBF, DC and CBF/DC ratio in several regions. Moreover, lower CBF/DC ratio in the left lingual gyrus negatively correlated with naming scores, lower CBF/DC ratio in medial frontal cortex/temporal gyrus positively correlated with the Montreal Cognitive Assessment (MoCA), visuospatial/executive and delayed recall scores.

**Conclusion:**

These findings may provide new potential insights into neuropathogenesis of cognition decline in stable COPD patients.

## Introduction

Chronic obstructive pulmonary disease (COPD) is the most common chronic lung disease in the general population, and is manifested with chronic irreversible airway limitation. The prevalence rate of COPD is about 13.6% in China (Fang et al., 2018). With the increase of age more significant than 40, the mortality rate of chronic respiratory diseases showed the most apparent increasing trend. Notably, cognitive impairment is widespread in patients with COPD (Charbek et al., 2019). The prevalence of cognitive impairment is reported to be about 16%–85% in patients with COPD (Simargi et al., 2022; Yohannes et al., 2017). Furthermore, COPD increases the risk of cognitive impairment by about 2.5 times (Dodd, Getov & Jones, 2010). However, the pathophysiological mechanism underlying cognitive impairment in COPD remains elusive.

fMRI techniques have been widely used to investigate the cerebral dysfunctions in COPD patients and provided evidences of the cerebral structural and functional abnormalities and their potential neural associations with cognitive impairment. These changes included gray matter atrophy, abnormal brain activity and disrupted white matter integrity of brain regions relevant for the cognition, including the frontal cortex, cingulate cortex, anterior insula, and hippocampus et al. (Chen et al., 2016; Li et al., 2020; Wang et al., 2017; Yu et al., 2016; Zhang et al., 2016), which can partly explain the pathophysiological and psychological changes in COPD patients.

CBF is defined as the delivery rate of arterial blood to the capillary bed in the cerebral tissue. Without complex confounding factors, resting state CBF is relatively easy to conduct, and is closely coupled with brain metabolism, including glucose utilization, oxygen consumption, and aerobic glycolysis (Vaishnavi et al., 2010). The arterial spin labeling (ASL), with good reliability and reproducibility (Sigurdsson et al., 2015), has been validated against other perfusion methods, and has been utilized to measure CBF in many studies (Lan et al., 2019; Thamm et al., 2019). Previous studies have shown that cerebral blood flow (CBF) coupled with cerebral metabolism and oxygen consumption (Fan et al., 2022; Jezzard, Chappell & Okell, 2018; Liu & Brown, 2007). Furthermore, studies from Varkuti et al. (2011) and Hagmann et al. (2008) also identified positive correlation between cerebral structural hubs and CBF. In addition, degree centrality (DC) is generally measured by extracting the time series of one voxel, correlating it with the time series of all the other voxels in the brain, and then calculating the summation of the resulting correlation coefficients. The DC could represent functional relationships between a voxel or region and the rest within the entire cerebral connectivity matrix (connectome) at the voxel level without requiring a priori selection. Neurovascular coupling refers to the correlation between CBF and neuronal activity and metabolism in the brain region, is a marker of cerebral function (Vestergaard et al., 2016). A study has indicated a correlation between the CBF measured with ASL and functional connectivity measured with BOLD in several networks (Liang et al., 2013). The CBF-DC correlation represents the consistency of spatial distribution between blood supply and functional hubs. The CBF/DC ratio represents the cerebral blood supply per unit of connectivity hub, reflecting the neurovascular coupling. The two indices could be used to identify changes in the neurovascular coupling in COPD that cannot be detected by investigating the CBF and DC separately. As far as we know, very few studies have directly examined the relationship between functional network hubs and the cerebral blood supply in COPD patients. Therefore, it is unknown that whether resting state intrinsic functional network connectivity is closely related to CBF and whether cerebral neurovascular changes involve the potential mechanisms underlying cognitive impairment of COPD patients.

In our study, resting-state BOLD fMRI and ASL data of COPD patients and healthy subjects were acquired during resting state. resting-state BOLD fMRI data were used to identify functional hubs in the brain, and ASL data were exploited to measure CBF. The CBF-DC coupling and CBF/DC ratio were compared between the two groups, and the potential correlations between functional deficits and clinical characteristics were also investigated. In the present study, we hypothesized that abnormal cerebral neurovascular alterations may contribute to cognitive deficits in COPD patients.

## Materials & Methods

### Participants

A total of eighty-five right-handed subjects were enrolled into this study, including forty-five patients with stable COPD and forty age- and gender-matched healthy controls. The patients with stable COPD were recruited from the Respiratory departments from March 2018 to April 2019.

Inclusion criteria for COPD group were as follows: (1) diagnosed according to pulmonary function test (PFT) (Singh et al., 2019); (2) PFT findings indicating: FEV1/FVC <0.70, 30% ≤ FEV_1_ ≤ 80% predicted; (3) stable stage is defined as the absence of exacerbation (defined as hospital admission or prescription of antibiotics/systemic corticosteroids by a general practitioner) in the past six weeks (as same as the study of our prior study Wang et al., 2020). Exclusion criteria for all subjects were: (1) home O_2_ therapy; (2) other pulmonary diseases, neurological disease except cognition decline, history of other cardiovascular and metabolic diseases, psychiatric disorder known to affect cognition; (3) alcohol/substance abuse or dependence; (4) medicine, drinking or smoking within 24 h before MRI examination; (5) body mass index >30 kg/m^2^; (6) any contraindication to fMRI examination. The healthy controls with comparable age, sex and education level were asymptomatic, free from a known history of cerebrovascular accident, heart failure psychiatric or neurologic disorders, obstructive sleep apnea, any metabolic disease, or other diseases known to affect cognition, and they were taking no medications.

Each subject underwent a physical examination and pulmonary function test. The Medical Research Council (MRC) Dyspnea Scale was used to quantify dyspnea’s degree (Fletcher, 1960). Montreal Cognitive Assessment (MoCA) was used for cognitive screening. COPD severity was also assessed by using the GOLD stage. The protocol was approved by the ethics committee of the Changzheng Hospital (2018SL028). Our work was carried out following the Declaration of Helsinki.

### Data acquisition

MRI examinations were performed at a 3T MR scanner (General Electrics) with an 8-channel head coil during resting state. 3D pseudo-continuous ASL images were acquired with the following parameters: time repetition/time echo = 4632/10.5 ms, slice thickness = four mm, the field of view = 240 × 240 mm^2^, NEX = 3.00, post-labeling delay=1525 ms. resting-state BOLD fMRI images were also acquired (time repetition /time echo = 2000/35 ms, flip angle = 90°, resolution = 64 × 64, field of view = 240 × 240 mm^2^, slice thickness = four mm, spacing = 0, slices = 38, 200 volumes). A high-resolution structural T1-weighted scan was performed with the following parameters: time repetition /time echo = 8.2/3.2 ms, flip angle = 12°, resolution = 256 × 256, slice thickness = one mm. Routine MRI examination images of all participants were acquired and checked by two experienced neuroradiologists to rule out anatomic abnormalities in the brain. For the resting state, all participants were instructed to relax and keep their eyes closed without thinking of anything in particular or falling asleep during scanning.

### Data processing of CBF and DC

The processing steps were performed by using the MATLAB2016 (MathWorks, Natick, MA, USA) platform. CBF images underwent the following preprocessing steps, including normalization into the standard Montreal Neurological Institute (MNI) space, resampling into a three mm voxel size, smoothing with a 6 mm^3^ full-width-at-half maximum Gaussian kernel (FWHM) and removing signal of white matter and cerebrospinal fluid.

The resting-state BOLD fMRI images processing steps included format conversion, removing first ten time points, slice timing, realign estimation, spatially coregistration, normalization into the standard MNI space, resampling into a three mm voxel size, band filtering (0.01−0.08 Hz) and linear detrend. Several spurious variances (white matter, cerebrospinal fluid, head motion and global mean signals) were removed via linear regression. Data were discarded if the translation exceeded two mm or if rotation exceeded 2°. Using the Resting-State fMRI Data Analysis Toolkit (REST version 1.8) software (Song et al., 2011), the voxel-wise DC was computed with *r* (correlation threshold) set at 0.25. Only positive weighted Pearson’s correlation coefficients were considered in this study.

### Voxel-Wise Comparisons in CBF and DC

By using REST software, the DC maps were *z*-transformed and smoothed (6 × 6 × 6 mm^3^ FWHM) to compare two groups. The intergroup comparisons of CBF and DC were performed voxel-wise while controlling for age, gender and education level (*q* < 0.05, FDR corrected).

### Region of interest (ROI) analysis

CBF maps were normalized into *z*-scores within the whole grey mask to improve normality. The regions obtained from voxel-wise comparisons of CBF and DC were merged and defined as regions of interest (ROIs). The CBF and DC values of each voxel in the merged ROIs were extracted for each participant. Furthermore, correlational analyses between CBF and DC were performed for each ROI of each participant. There is a CBF-DC coefficient value for each ROI of each participant, reflecting the consistency of spatial distribution between CBF and DC at the ROI level. Then, a two-sample *t* test was used to compare the difference in CBF-DC correlation coefficients between the two groups.

To evaluate the amount of blood supply per unit of functional hubs, the CBF/DC ratio (both CBF and DC were original values without *z*-transformation) of each voxel were calculated and transformed into a *z*-score map to improve the normality. Based on the merged ROI, differences in CBF/DC ratio betw‘een the two groups were analyzed. For each participant, the mean CBF/DC ratio of each ROI was extracted by using REST software.

### Statistical analysis

Two-sample *t* tests were performed to examine the differences in age, education and clinical symptoms scores between the two groups using SPSS 21.0 (SPSS Inc., Chicago, IL, USA), while Chi square test to analyze differences in gender. The statistical significance of group differences was set at *p* < 0.05. Comparisons of fMRI index (CBF, DC, CBF-DC coupling and CBF/DC ratio) were performed as methods abovementioned. The brain networks were visualized with the BrainNet Viewer (http://www.nitrc.org/projects/bnv/) (Xia et al., 2013). Furthermore, A partial correlation analysis was performed to identify the association between CBF, DC, CBF/DC ratio and clinical characteristics (lung test index, MRC dyspnea severity, MoCA), with age, gender and education level as nuisance covariates; results with *q* < 0.05, FDR-corrected were considered statistically significant.

## Results

### Clinical characteristics differences

No significant differences were found in age (*t* =  − 0.735, *df* = 83, *p* = 0.465), gender (*χ2* = 0.262, *df* = 1, *p* = 0.609) and BMI (*t* = 1.989, *df* = 83, *p* = 0.667) between the COPD and control groups. Compared to HC, COPD showed lower lung function in FEV_1_% predicted (*t* =  − 14.142, *df* = 83, *p* < 0.001) and FEV1/FVC % predicted (*t* =  − 13.972, *df* = 83, *p* < 0.001). COPD patients showed lower MoCA (*t* =  − 13.905, *df* = 83, *p* < 0.001) and subdomain scores (*p* < 0.05). Details were shown in Table 1.

**Table 1:** Demographic and clinical data by group.

	Group; mean (SD)				
Characteristics	COPD	Control	df	*p* value	*t*/*χ2*
Number of subjects	45	40		–	–
Age (years)	67.93 (7.06)	69.15 (6.15)	83	0.465	−0.735^*^
Age rang (years)	52∼79	53∼78		–	–
Education (years)	11.28 (3.39)	11.78 (3.35)	83	0.581	−0.555
Gender (Male/Female)	25/20	20/20	1	0.609	0.262^*^
Body Mass Index (kg/m^2^)	23.3 (3.1)	23 (3.3)	83	0.667	1.989
Duration of disease (years)	7.2 (5.12)	N/A		–	–
Number of Smokers	23	19	1	0.74	0.11^*^
FEV1/FVC (%predicted)	58.63 (11.47)	85.18 (3.81)	83	<0.001	−13.972
FEV1 (%predicted)	54.78 (16.40)	96.68 (9.59)	83	<0.001	−14.142
MoCA	18.27 (4.68)	28.78 (1.03)	83	<0.001	−13.905
Visuospatial/Executive	2.2 (1.32)	4.68 (0.57)	83	<0.001	−10.939
Naming	2.18 (0.68)	2.83 (0.55)	83	0.011	−4.77
Attention	4.31 (1.47)	5.75 (0.49)	83	<0.001	−5.883
Language	2.2 (0.94)	2.85 (0.36)	83	<0.001	−4.094
Abstraction	1.49 (0.63)	1.93 (0.27)	83	<0.001	−4.086
Delayed Recall	1.51 (1.25)	4.93 (0.27)	83	<0.001	−16.865
Orientation	4.38 (1.89)	5.88 (0.34)	83	<0.001	−4.948
Dyspnea (mMRC)	2.31 (0.51)	N/A			

**Notes.**

COPD chronic obstructive pulmonary disease FEV1 forced expiratory volume in 1 second (#8175) FEV1/FVC forced expiratory volume in 1 s/ forced vital capacity mMRC modified Medical Research Council scale for dyspnea MoCA Montreal Cognitive Assessment N/A Not applicable SD standard deviation

* Chi square test, while others were tested by student’s *t* test.

### Differences in CBF and DC

**Figure 1:**
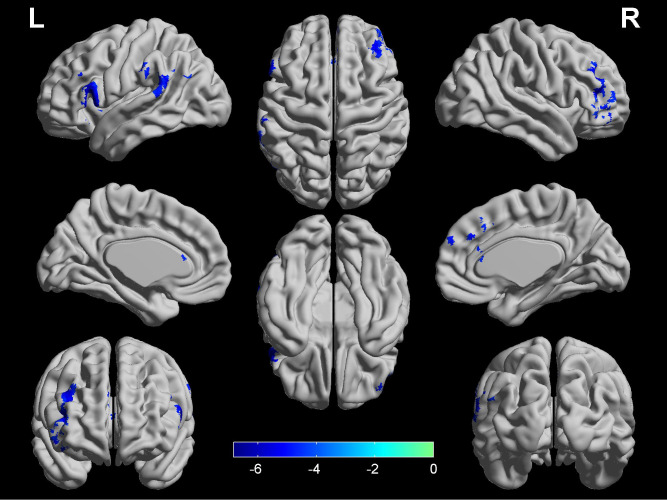
Significant differences in CBF between COPD patients and HC. Four regions (left DLPFC, right DLPFC, left SMG and bilateral ACG) show lower CBF in COPD patients (*q* < 0.05, FDR corrected) while controlling for the age, gender and education level. The color bar represents the *t* value from two-sample *t*-test. ACG, anterior cingulum gyrus; CBF, cerebral blood flow; COPD, chronic obstructive pulmonary disease; LPFC, dorsolateral prefrontal cortex; HC, healthy controls; SMG, supramarginal gyrus; L, left; R, right.

**Figure 2:**
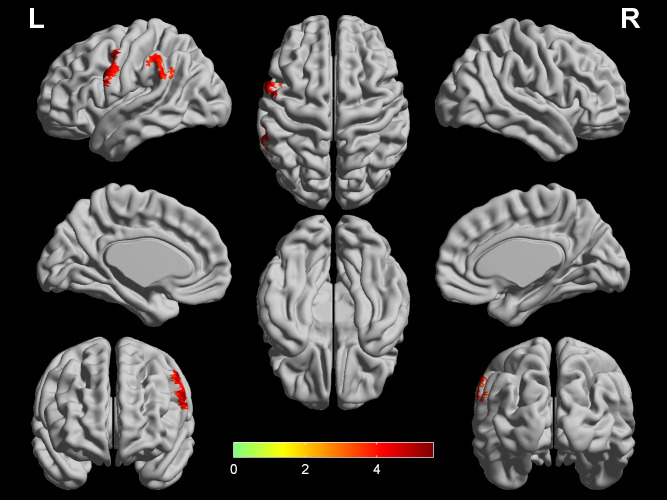
Significant differences in DC between COPD patients and HC. Two regions (left PreCG and left SMG) show higher DC in COPD patients (*q* < 0.05, FDR corrected) while controlling for the age, gender and education level. The color bar represents the *t* value from two-sample *t*-test. CBF, cerebral blood flow; COPD, chronic obstructive pulmonary disease; HC, healthy controls; PreCG, precentral gyrus; SMG, supramarginal gyrus; L, left; R, right.

As depicted in Fig. 1, the COPD group showed decreased CBF in the left dorsolateral prefrontal cortex (DLPFC), right DLPFC, left supramarginal gyrus (SMG) and bilateral anterior cingulum gyrus (ACG). Further correlational analyses showed no significant correlations between any regions with CBF and any clinical variables in the COPD group (*p* > 0.05). As depicted in Fig. 2, the COPD group showed greater DC in the left precentral gyrus (PreCG) and left SMG. The mean DC values in the left SMG showed significantly negative correlation with MoCA score (*r* =  − 0.478, *p* = 0.001) and visuospatial/executive score (*r* =  − 0.574, *p* < 0.001), with age, gender, education level as covariates, and FDR corrected for multiple comparisons. Details were shown in Table 2.

### CBF-DC coupling analysis

The merged maps with significantly intergroup clusters in CBF and DC comparisons covered four brain clusters, including the left PreCG/DLPFC, right DLPFC, left SMG and bilateral ACG. The CBF has significantly correlated DC in the four merged regions in all participants. There were no significant intergroup differences of the correlation coefficients in the left PreCG/PLPFC (*t* = 1.59, *df* = 83, *p* = 0.12), right DLPFC (*t* =  − 0.72, *df* = 83, *p* = 0.48), left SMG (*t* =  − 1.04, *df* = 83, *p* = 0.30) and bilateral ACG (*t* =  − 1.84, *df* = 83, *p* = 0.07).

### Comparison of CBF-DC ratio

**Figure 3:**
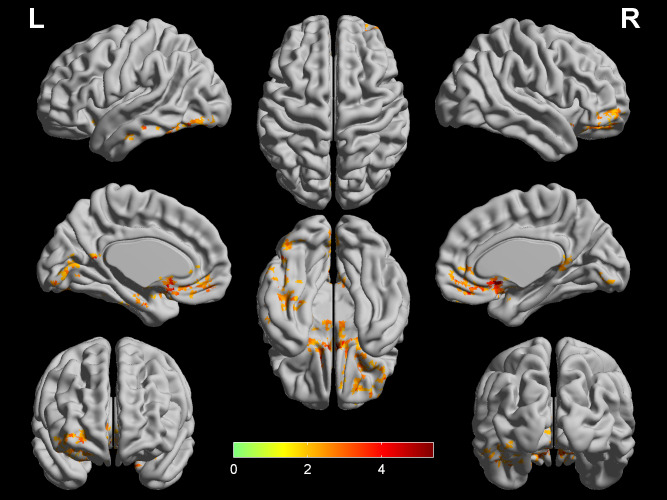
Comparison of CBF/DC ratio between COPD patients and HC. Two regions (bilateral media frontal cortex/temporal gyrus and left supramarginal gyrus) show higher CBF/DC ratio in COPD patients (*q* < 0.05, FDR corrected) while controlling for the age, gender and education level. The color bar represents the *t* value from two-sample *t*-test. CBF, cerebral blood flow; COPD, chronic obstructive pulmonary disease; HC, healthy controls.

**Figure 4:**
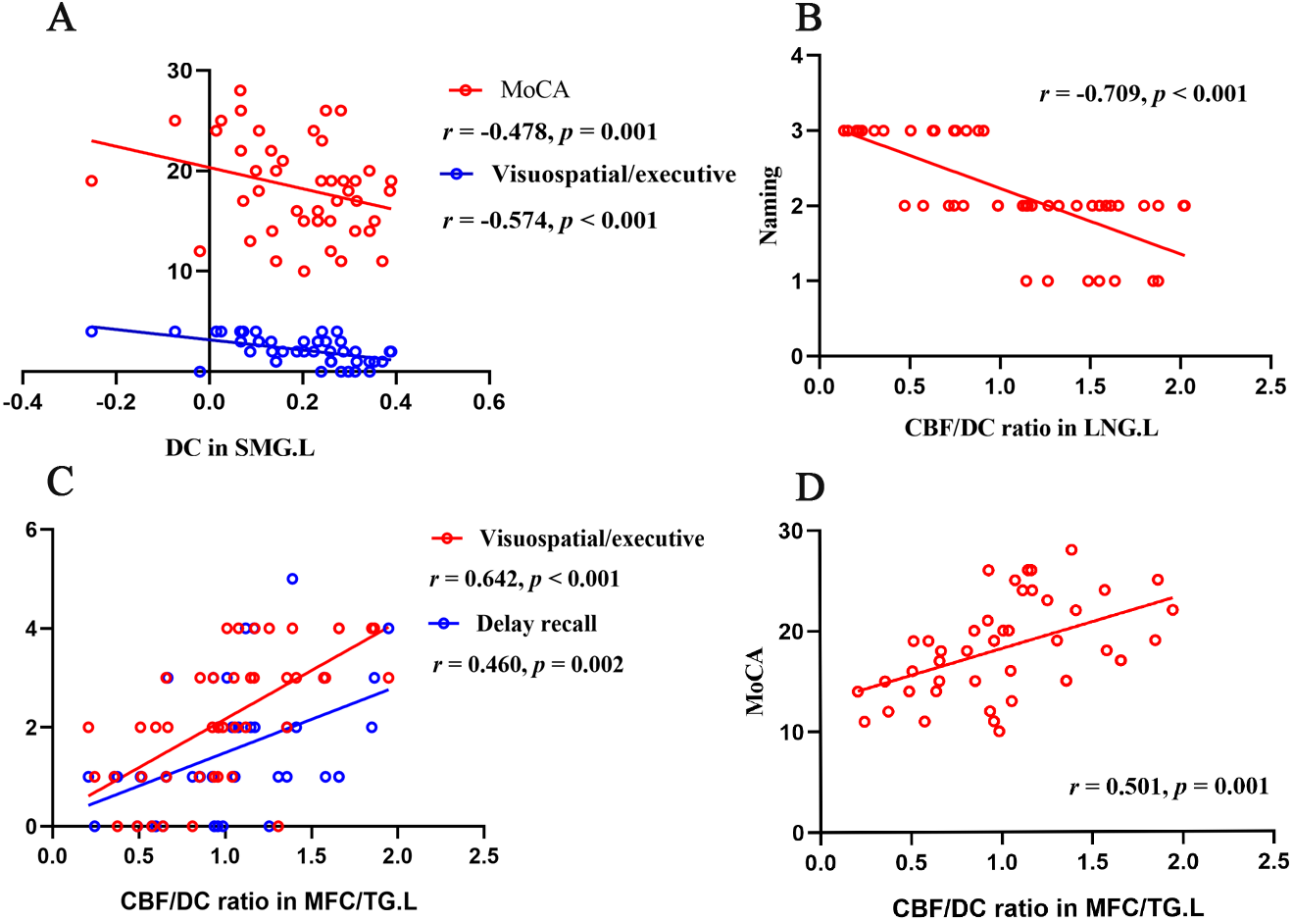
Correlation analyses of functional parameters and clinical variates. (A) DC in left SMG negatively correlated with MoCA score ( *r* =  − 0.478, *p* = 0.001) and visuospatial/executive score ( *r* =  − 0.574, *p* < 0.001); (B) CBF/DC ratio in left LNG negatively correlated with naming score (*r* =  − 0.709, *p* < 0.001); (C) CBF/DC ratio in MFC/TG.L positively correlated with visuospatial/executive score (*r* = 0.642, *p* < 0.001) and delay recall score (*r* = 0.460, *p* = 0.002); (D) CBF/DC ratio in MFC/TG.L positively correlated with MoCA (*r* = 0.501, *p* = 0.001). CBF, cerebral blood flow; COPD, chronic obstructive pulmonary disease; HC, healthy controls; LNG, lingual gyrus; MFC, medial frontal gyrus; SMG, supramarginal gyrus; TG, temporal gyrus; L, left; R, right.

At the voxel level, the COPD group showed increased CBF/DC ratio in the bilateral medial frontal cortex (ventromedial prefrontal cortex, orbitofrontal cortex and rectus gyrus), bilateral caudate nucleus, left middle temporal gyrus (parahippocampus gyrus and fusiform gyrus), and left lingual gyrus (LNG), seen in Fig. 3. Furthermore, in the COPD group, the correlational analyses revealed that the CBF/DC ratio in the left LNG showed significantly negative correlations with the naming score (*r* =  − 0.709, *p* < 0.001), and the CBF/DC ratio in the medial frontal cortex/temporal gyrus showed significantly positive correlations with MoCA score (*r* = 0.501, *p* = 0.001), visuospatial/executive score (*r* = 0.642, *p* < 0.001) and delayed recall score (*r* = 0.460, *p* = 0.002), with age, gender, education level as covariates, and FDR corrected for multiple comparisons, seen in Fig. 4. Furthermore, the mean CBF and DC values in the above regions were extracted for intergroup comparison. In the left LNG, COPD patients showed lower CBF (*t* =  − 2.174, *df* = 8, *p* = 0.035) and lower DC (*t* =  − 2.610, *df* = 83, *p* = 0.012) than the healthy controls; lower CBF (*t* =  − 3.744, *df* = 83, *p* = 0.001) and lower DC (*t* =  − 3.818, *df* = 83, *p* < 0.001) were also observed in the medial frontal cortex/temporal gyrus in COPD group.

In ROI analyses, compared to healthy controls, COPD patients exhibited decreased CBF/FCS ratio in the left PreCG/DLPFC (*t* =  − 2.711, *df* = 83, *p* = 0.01) and left SMG (*t* =  − 2.452, *df* = 83, *p* = 0.018). However, no significant correlation between the CBF/DC ratio of any ROI and any clinical characteristics was found in the COPD group. Further validation of CBF and DC changes showed that COPD patients exhibited lower CBF and higher DC in the two regions (*p* < 0.001).

## Discussion

As far as we know, this is the first study to use both resting-state CBF and DC approaches to investigate cerebral neurovascular coupling changes related to the potential neurological mechanisms underlying cognitive impairment in stable COPD patients. Compared with healthy controls, COPD patients showed decreased CBF in the bilateral DLPFC, ACG and left SMG, and increased DC in the left PreCG and left SMG. More importantly, COPD showed increased CBF/DC ratio in the bilateral medial frontal cortex, bilateral caudate nucleus, left temporal gyrus and left LNG, and CBF/DC ratio in several brain regions significantly correlated with MoCA, visuospatial/executive and delay recall scores. These findings may provide neuroimaging evidence and improve our understanding of neural mechanisms underlying cognitive impairment in COPD patients from the perspective of neurovascular coupling.

**Table 2:** Comparisons of CBF, DC between COPD and HC.

Brain region	MNI Coordinates			Number of Voxels	Peak *t* value
	x	y	z		
***CBF***					
DLPFC.L	−57	21	12	167	−6.839
DLPFC.R	39	39	12	176	−5.923
SMG.L	-66	-33	24	113	−5.827
ACG.B	3	36	30	90	−5.161
***DC***					
PreCG.L	−60	9	33	130	5.81
SMG.L	−63	−51	39	105	4.508

**Notes.**

ACG anterior cingulum gyrus CBF cerebral blood supply COPD chronic obstructive pulmonary disease DC degree centrality DLPFC dorsolateral prefrontal cortex HC healthy control PreCG precentral gyrus SMG supramarginal gyrus L left R right B bilateral

Voxel-wise analyses revealed that COPD patients showed higher CBF/DC ratio in several regions. Subsequent ROI-based analyses revealed lower CBF and lower DC values of these regions in the COPD patients. But, by using voxel-wise analyses, there was no overlap between the merged regions generated in intergroup comparisons of CBF, DC and regions with intergroup different CBF/DC ratio; we speculated that CBF/DC ratio could enlarge the differences between the COPD and healthy group. Moreover, COPD patients showed a significantly decreased CBF/DC ratio in some merged ROIs. We demonstrated that a combination of CBF, DC and CBF/DC ratio based on voxel- and ROI-wise analyses might be a comprehensive and reliable method to explore the underlying mechanism of COPD patients. In the present study, COPD patients showed increased CBF/DC ratio in the left LNG, which was driven by disproportionally attenuated CBF and DC. The LNG involves in encoding of complex images (Machielsen et al., 2015), identification and recognition of words (Mechelli et al., 2000). Supporting this, the LNG is functionally associated with decreased naming performance (Deverdun et al., 2019) and visual processing (Chee et al., 2009) and visual hallucination (Goldman et al., 2014). In the COPD group, the negative correlation between the higher CBF/DC ratio of the LNG and the naming score indicated that the attenuation degree of DC is greater than that of CBF in this region, resulting in decompensated increase of CBF per unit of functional connectivity in the left LNG and poor naming function. In addition, COPD patients also showed higher CBF/DC ratio in the bilateral medial frontal cortex, bilateral caudate nucleus, and left middle temporal gyrus. Several COPD studies have provided evidence of reduced thickness and volume in these regions (Chen et al., 2016; Zhang et al., 2013). Increased CBF/DC ratio of these regions may play a compensatory role for the reduced grey matter volume. In the present study, the increased CBF/DC ratio in bilateral medial frontal cortex and left middle temporal gyrus is driven by decreased CBF and DC. Consist with our study, hypoperfusion in the frontal cortex and cognitive abnormalities have been found in COPD patients (Hulya & Seniha, 2006). Based on the finding of positive correlation between higher CBF/DC ratio in this region and MoCA, visuospatial/executive and delayed recall. A possible explanation for these increased CBF-DC ratio could be the compensation mechanism that acts to counterbalance regional deficits in cognitive function. Compensatory mechanisms accompany the cognitive impairments progress in COPD patient. In addition, synaptic loss is associated with cognitive decline and compensatory mechanisms, which can alleviate cognitive impairment caused by synaptic loss by maintaining the activity level of neural circuits. Here, the finding of increased CBF-DC ratio in COPD suggests that COPD patients could use additional blood supply per function hub unit for cognitive function, presumably suggesting the plasticity of human brain compensating for cognition decline.

In the ROI analyses, COPD patients exhibited decreased CBF/DC ratio in the left PreCG/DLPFC and left SMG which involved in somatic motor function and spatial working memory. Decreased grey matter density and neural activation in the left PreCG have been reported in COPD patients (Zhang et al., 2012; Zhang et al., 2013; Zhang et al., 2016). The DLPFC is a crucial part of dorsal visual processing stream regions involved in visual reproduction impairment in COPD. A surface-based morphometry study suggested that the thinner DLPFC is a predictive factor of poorer visual reproduction performance (Chen et al., 2016). They also found reduced cortical thickness and surface in the PreCG and SMG in COPD. In our study, these regions showed lower CBF and higher DC, suggesting that the decreased CBF/DC ratios were driven by the CBF decrease and DC increase, which might relate to reduced cortical thickness and surface, rendering to cognitive impairment in COPD.

In voxel-wise analyses, several brain regions showed significant intergroup differences in CBF and DC between COPD patients and healthy controls. The brain constitutes only about 2% of the body weight, but easily receives up to 15–20% of the total cardiac output as CBF. CBF changes may be ascribed to the following reasons. The hypothesis of neurovascular coupling suggested that neural activity changes govern CBF through complex coordinated mechanisms involving neurons, glial cells, and vascular components (Venkat, Chopp & Chen, 2016). Moreover, the neural stimuli may be involved in controlling the diameter of the cerebral vessel and brain blood supply, resulting in CBF changes. Finally, chemical mediators, such as neuroinflammation factors, adenosine, nitric oxide (Lourenço et al., 2014), hydrogen, potassium, calcium and lactate (Dienel, 2012), may trigger hemodynamic responses resulting in vasodilation/vasoconstriction and CBF changes. In the present study, COPD patients showed decreased CBF in bilateral DLPFC, ACG and left SMG. Meanwhile, higher DC values were found in the left PreCG and left SMG in COPD. The higher DC represents more correlations between the given voxel and the rest voxels, indicating that neurons in this voxel are more critical and active. Tomasi & Volkow (2010) suggested that voxels with high DC serve as the interconnection hubs, meaning effective and fast brain communication with minimal energy cost. Widespread evidence of increased functional activation has been reported in patients with COPD (Dodd et al., 2012; Xin et al., 2019; Yu et al., 2016; Zhang et al., 2016). Increased resting state connection could be interpreted as reducing precise control over functional networks that are not beneficial, indicating a disrupt network. DLPFC is a region involved in visual reproduction and spatial working memory (Courtney et al., 1998), which is sensitive to hypoxia (Jayalakshmi et al., 2007). Furthermore, Chen et al. (2016) suggested that the thinner DLPFC is a predictive factor of poorer visual reproduction performance. And Kravitz et al. demonstrated that DLPFC is a key neural locus for short-term visual memory (Kravitz et al., 2011). Previous dyspnea-related fMRI studies had reported activation in the ACG (Herigstad et al., 2015). In addition, by using breathlessness-related word-cue task, another task study showed that baseline activity in the ACG and prefrontal cortex correlated with improvements in breathlessness and breathlessness-anxiety (Herigstad et al., 2017). Moreover, the SMG is involving in a multimodal complex that integrates somatosensory inputs to the brain and is associated with attention processing. Meanwhile, higher DC values were found in the left PreCG and left SMG in COPD. The higher DC represents more correlations between the given voxel and the rest voxels, indicating that neurons in this voxel are more critical and active. Tomasi & Volkow (2010) suggested that voxels with high DC serve as the interconnection hubs, meaning effective and fast brain communication with minimal energy cost. Widespread evidence of increased functional activation has been reported in patients with COPD (Dodd et al., 2012; Xin et al., 2019; Yu et al., 2016; Zhang et al., 2016). Increased resting state connection could be interpreted as reducing precise control over functional networks that are not beneficial, indicating a disrupt network. Our study showed a significantly negative correlation between the mean DC value of the left supramarginal gyrus and the visuospatial/executive function score, suggesting that the left SMG may be associated with visuospatial/executive dysfunction in COPD patients. Taken together, the CBF and DC DLPFC, ACG, SMG and PreCG were impaired in COPD patients, which might provide new neuroimaging evidence contributing to the neural basis of cognitive impairment in COPD patients.

We found significant correlations between CBF and DC in the four merged ROIs in both COPD and healthy groups. Further comparison analyses showed no significant differences in the correlation coefficients between the two groups which meant normal neurovascular coupling in the four ROIs in all subjects. To minimize interference factors, we excluded participants with other diseases or disorders that may affect the cerebral function and structure, such as cardiovascular, metabolic diseases, neurosis and psychosis. We speculated that this finding might be associated with current status of COPD patients in this study. More COPD patients with different stages may be needed to explore in the further studies. Unexpectedly, no associations between abnormal brain functions and pulmonary-specific disease markers were found. We conceived that the extrapulmonary manifestations of COPD may not be strongly related to pulmonary-specific disease markers. In addition, a large body of literature provided evidence that cigarette smoking involves in the cerebral functional or structural abnormalities in COPD (Baeza-Loya et al., 2016; Dodd et al., 2012; Gons et al., 2011; Taki et al., 2013). However, no correlation between the duration or the amount of smoking and the cerebral abnormalities were found in all smokers.

Several limitations should be taken into account when interpreting our findings. Firstly, relatively small sample size may influence our interpretations. More COPD patients are needed in the further investigations. Secondly, CBF and DC are indirect indices, preventing us from direct and more reliable measurements of cerebral perfusion and neural activity. Thirdly, analysis of grey matter volume should be performed to further support our present findings. Finally, COPD patients after oxygen therapy are not enrolled in this study, a follow-up investigation with longitudinal comparison is needed to validate the present findings.

## Conclusion

Our study revealed disrupted neurovascular coupling in COPD patients via a combination of resting-state BOLD fMRI and ASL techniques. Specifically, increased CBF/DC ratio in the left lingual gyrus, medial frontal cortex/temporal gyrus were involved in cognitive processing in COPD patients. These findings presented novel evidence that abnormal neurovascular coupling may contribute to a potential neural mechanism of cognitive impairment in COPD patients.

##  Supplemental Information

10.7717/peerj.14249/supp-1 Data S1 Data of Table1 & Figure 4

## Additional Information and Declarations

### Data Availability

The following information was supplied regarding data availability: The raw data for demographic & clinical data are available in the Supplemental File.

## References

[ref-1] Baeza-Loya S, Velasquez KM, Molfese DL, Viswanath H, Curtis KN, Thompson-Lake DG, Baldwin PR, Ellmore TM, De La Garza 2nd R, Salas R (2016). Anterior cingulum white matter is altered in tobacco smokers. The American Journal on Addictions.

[ref-2] Charbek E, Huynh K, Kim E, Nayak RP (2019). Assessment of cognitive impairment in patients with chronic obstructive pulmonary disease using the rapid cognitive screen. The Journal of Nutrition, Health and Aging.

[ref-3] Chee MW, Chen KH, Zheng H, Chan KP, Isaac V, Sim SK, Chuah LY, Schuchinsky M, Fischl B, Ng TP (2009). Cognitive function and brain structure correlations in healthy elderly East Asians. Neuroimage.

[ref-4] Chen J, Lin I, Zhang H, Lin J, Zheng S, Fan M, Zhang J (2016). Reduced cortical thickness, surface area in patients with chronic obstructive pulmonary disease: a surface-based morphometry and neuropsychological study. Brain Imaging and Behavior.

[ref-5] Courtney SM, Petit L, Maisog JM, Ungerleider LG, Haxby JV (1998). An area specialized for spatial working memory in human frontal cortex. Science.

[ref-6] Deverdun J, Van Dokkum LEH, Le Bars E, Herbet G, Mura T, D’agata B, Picot M-C, Menjot N, Molino F, Duffau H, Moritz Gasser S (2019). Language reorganization after resection of low-grade gliomas: an fMRI task based connectivity study. Brain Imaging and Behavior.

[ref-7] Dienel GA (2012). Brain lactate metabolism: the discoveries and the controversies. Journal of Cerebral Blood Flow & Metabolism.

[ref-8] Dodd JW, Chung AW, Broek MDVanden, Barrick TR, Charlton RA, Jones PW (2012). Brain structure and function in chronic obstructive pulmonary disease: a multimodal cranial magnetic resonance imaging study. American Journal of Respiratory and Critical Care Medicine.

[ref-9] Dodd JW, Getov SV, Jones PW (2010). Cognitive function in COPD. European Respiratory Journal.

[ref-10] Fan D, He C, Liu X, Zang F, Zhu Y, Zhang H, Zhang H, Zhang Z, Xie C (2022). Altered resting-state cerebral blood flow and functional connectivity mediate suicidal ideation in major depressive disorder. Journal of Cerebral Blood Flow & Metabolism.

[ref-11] Fang L, Gao P, Bao H, Tang X, Wang B, Feng Y, Cong S, Juan J, Fan J, Lu K, Wang N, Hu Y, Wang L (2018). Chronic obstructive pulmonary disease in China: a nationwide prevalence study. The Lancet Respiratory Medicine.

[ref-12] Fletcher CM (1960). Standardised questionnaire on respiratory symptoms: a statement prepared and approved by the MRC Committee on the Aetiology of Chronic Bronchitis (MRC breathlessness score). BMJ.

[ref-13] Goldman JG, Stebbins GT, Dinh V, Bernard B, Merkitch D, De Toledo-Morrell L, Goetz CG (2014). Visuoperceptive region atrophy independent of cognitive status in patients with Parkinson’s disease with hallucinations. Brain.

[ref-14] Gons RA, Van Norden AG, De Laat KF, Van Oudheusden LJ, Van Uden IW, Zwiers MP, Norris DG, De Leeuw FE (2011). Cigarette smoking is associated with reduced microstructural integrity of cerebral white matter. Brain.

[ref-15] Hagmann P, Cammoun L, Gigandet X, Meuli R, Honey CJ, Wedeen VJ, Sporns O (2008). Mapping the structural core of human cerebral cortex. PLOS Biology.

[ref-16] Herigstad M, Faull OK, Hayen A, Evans E, Hardinge FM, Wiech K, Pattinson KTS (2017). Treating breathlessness via the brain: changes in brain activity over a course of pulmonary rehabilitation. European Respiratory Journal.

[ref-17] Herigstad M, Hayen A, Evans E, Hardinge FM, Davies RJ, Wiech K, Pattinson KTS (2015). Dyspnea-related cues engage the prefrontal cortex: evidence from functional brain imaging in COPD. Chest.

[ref-18] Hulya O, Seniha N (2006). Brain perfusion abnormalities in chronic obstructive pulmonary disease: comparison with cognitive impairment. Annals of Nuclear Medicine.

[ref-19] Jayalakshmi K, Singh SB, Kalpana B, Sairam M, Muthuraju S, Ilavazhagan G (2007). N-acetyl cysteine supplementation prevents impairment of spatial working memory functions in rats following exposure to hypobaric hypoxia. Physiology & Behavior.

[ref-20] Jezzard P, Chappell MA, Okell TW (2018). Arterial spin labeling for the measurement of cerebral perfusion and angiography. Journal of Cerebral Blood Flow & Metabolism.

[ref-21] Kravitz DJ, Saleem KS, Baker CI, Mishkin M (2011). A new neural framework for visuospatial processing. Nature Reviews Neuroscience.

[ref-22] Lan MJ, Rubin-Falcone H, Elizabeth Sublette M, Oquendo MA, Stewart JW, Hellerstein DJ, McGrath PJ, Zanderigo F, John Mann J (2019). Deficits of white matter axial diffusivity in bipolar disorder relative to major depressive disorder: no relationship to cerebral perfusion or body mass index. Bipolar Disorder.

[ref-23] Li H, Xin H, Yu J, Yu H, Zhang J, Wang W, Peng D (2020). Abnormal intrinsic functional hubs and connectivity in stable patients with COPD: a resting-state MRI study. Brain Imaging and Behavior.

[ref-24] Liang X, Zou Q, He Y, Yang Y (2013). Coupling of functional connectivity and regional cerebral blood flow reveals a physiological basis for network hubs of the human brain. Proceedings of the National Academy of Sciences of the United States of America.

[ref-25] Liu TT, Brown GG (2007). Measurement of cerebral perfusion with arterial spin labeling: Part 1. Methods. Journal of the International Neuropsychological Society.

[ref-26] Lourenço CF, Santos RM, Rui MB, Cadenas E, Radi R, Laranjinha J (2014). Neurovascular coupling in hippocampus is mediated via diffusion by neuronal-derived nitric oxide. Free Radical Biology and Medicine.

[ref-27] Machielsen WCM, Rombouts SARB, Barkhof F, Scheltens P, Witter MP (2015). fMRI of visual encoding: Reproducibility of activation. Human Brain Mapping.

[ref-28] Mechelli A, Humphreys GW, Mayall K, Olson A, Price CJ (2000). Contrasting effects of wordlength and visual contrast in fusiform and lingual gyri during reading. Neuroimage.

[ref-29] Sigurdsson S, Forsberg L, Aspelund T, Van der Geest RJ, Van Buchem MA, Launer LJ, Gudnason V, Van Osch MJ (2015). Feasibility of using pseudo-continuous arterial spin labeling perfusion in a geriatric population at 1.5 T. PLOS ONE.

[ref-30] Simargi Y, Mansyur M, Turana Y, Harahap AR, Ramli Y, Siste K, Prasetyo M, Rumende CM (2022). Risk of developing cognitive impairment on patients with chronic obstructive pulmonary disease: a systematic review. Medicine.

[ref-31] Singh D, Agusti A, Anzueto A, Barnes PJ, Bourbeau J, Celli BR, Criner GJ, Frith P, Halpin DMG, Han M, Lopez Varela MV, Martinez F, Montes De Oca M, Papi A, Pavord ID, Roche N, Sin DD, Stockley R, Vestbo J, Wedzicha JA, Vogelmeier C (2019). Global strategy for the diagnosis, management, and prevention of chronic obstructive lung disease: the GOLD science committee report 2019. European Respiratory Journal.

[ref-32] Song XW, Dong ZY, Long XY, Li SF, Zuo XN, Zhu CZ, He Y, Yan CG, Zang YF (2011). REST: a toolkit for resting-state functional magnetic resonance imaging data processing. PLOS ONE.

[ref-33] Taki Y, Kinomura S, Ebihara S, Thyreau B, Sato K, Goto R, Kakizaki M, Tsuji I, Kawashima R, Fukuda H (2013). Correlation between pulmonary function and brain volume in healthy elderly subjects. Neuroradiology.

[ref-34] Thamm T, Guo J, Rosenberg J, Liang T, Marks MP, Christensen S, Do HM, Kemp SM, Adair E, Eyngorn I, Mlynash M, Jovin TG, Keogh BP, Chen HJ, Lansberg MG, Albers GW, Zaharchuk G (2019). Contralateral hemispheric cerebral blood flow measured with arterial spin labeling can predict outcome in acute stroke. Stroke.

[ref-35] Tomasi D, Volkow ND (2010). Functional connectivity density mapping. Proceedings of the National Academy of Sciences of the United States of America.

[ref-36] Vaishnavi SN, Vlassenko AG, Rundle MM, Snyder AZ, Mintun MA, Raichle ME (2010). Regional aerobic glycolysis in the human brain. Proceedings of the National Academy of Sciences of the United States of America.

[ref-37] Varkuti B, Cavusoglu M, Kullik A, Schiffler B, Veit R, Yilmaz O, Rosenstiel W, Braun C, Uludag K, Birbaumer N, Sitaram R (2011). Quantifying the link between anatomical connectivity, gray matter volume and regional cerebral blood flow: an integrative MRI study. PLOS ONE.

[ref-38] Venkat P, Chopp M, Chen J (2016). New insights into coupling and uncoupling of cerebral blood flow and metabolism in the brain. Croatian Medical Journal.

[ref-39] Vestergaard MB, Lindberg U, Jacob Aachmann-Andersen N, Lisbjerg K, Just Christensen S, Law I, Rasmussen P, Olsen NV, Larsson HBW (2016). Acute hypoxia increases the cerebral metabolic rate—a magnetic resonance imaging study. Journal of Cerebral Blood Flow and Metabolism.

[ref-40] Wang C, Ding Y, Shen B, Gao D, An J, Peng K, Hou G, Zou L, Jiang M, Qiu S (2017). Altered gray matter volume in stable chronic obstructive pulmonary disease with subclinical cognitive impairment: an exploratory study. Neurotoxicity Research.

[ref-41] Wang W, Wang P, Li Q, Peng Z, Wang X, Wang G, Hou J, Fan L, Liu S (2020). Alterations of grey matter volumes and network-level functions in patients with stable chronic obstructive pulmonary disease. Neuroscience Letters.

[ref-42] Xia M, Wang J, Yong H, Peter C (2013). BrainNet viewer: a network visualization tool for human brain connectomics. PLOS ONE.

[ref-43] Xin H, Li H, Yu H, Yu J, Zhang J, Wang W, Peng D (2019). Disrupted resting-state spontaneous neural activity in stable COPD. International Journal of Chronic Obstructive Pulmonary Disease.

[ref-44] Yohannes AM, Chen W, Moga AM, Leroi I, Connolly MJ (2017). Cognitive impairment in chronic obstructive pulmonary disease and chronic heart failure: a systematic review and meta-analysis of observational studies. Journal of the American Medical Directors Association.

[ref-45] Yu L, De Mazancourt M, Hess A, Ashadi FR, Klein I, Mal H, Courbage M, Mangin L (2016). Functional connectivity and information flow of the respiratory neural network in chronic obstructive pulmonary disease. Human Brain Mapping.

[ref-46] Zhang H, Wang X, Lin J, Sun Y, Huang Y, Yang T, Zheng S, Fan M, Zhang J (2012). Grey and white matter abnormalities in chronic obstructive pulmonary disease: a case-control study. BMJ Open.

[ref-47] Zhang H, Wang X, Lin J, Sun Y, Huang Y, Yang T, Zheng S, Fan M, Zhang J (2013). Reduced regional gray matter volume in patients with chronic obstructive pulmonary disease: a voxel-based morphometry study. American Journal of Neuroradiology.

[ref-48] Zhang J, Chen J, Yu Q, Fan C, Zhang R, Lin J, Yang T, Fan M (2016). Alteration of spontaneous brain activity in COPD patients. International Journal of Chronic Obstructive Pulmonary Disease.

